# Supplementary material for: Identification of photoperiod-induced specific miRNAs in the adrenal glands of Sunite sheep (Ovis aries)
Source: Front Vet Sci. 2022 Jul 22;9:888207. doi: 10.3389/fvets.2022.888207 (PMC9354845; doi:10.3389/fvets.2022.888207)
Supplement: Supplementary file 1 [file Data_Sheet_1.ZIP › Supplementary file/miRDeep2 file.zip!/miRDeep2 file/Novel_Structure/LP42A3/Novel_603.pdf]

Provisional ID	:	NC_019464.2_9730
Score total	:	12.4
Score for star read(s)	:	3.9
Score for read counts	:	4.7
Score for mfe	:	2.2
Score for randfold	:	1.6
Score for cons. seed	:	
Total read count	:	21
Mature read count	:	19
Loop read count	:	0
Star read count	:	2

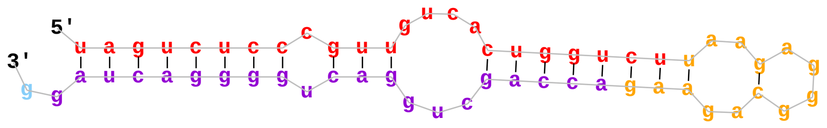

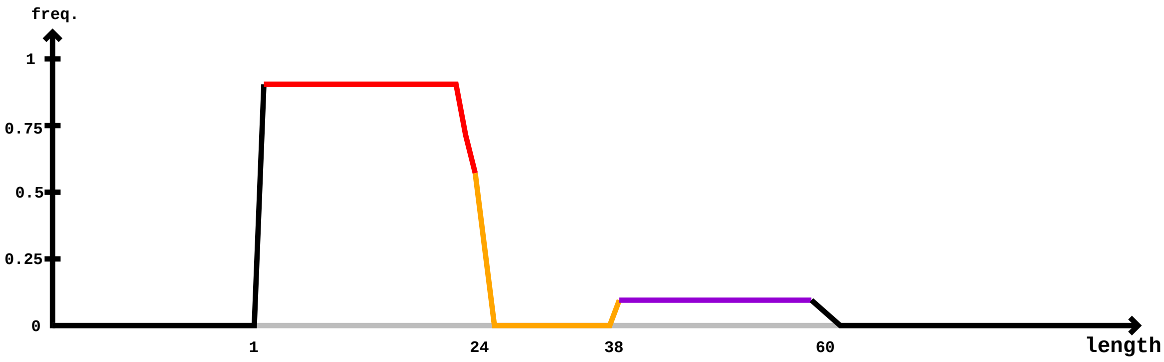

Mature **Star**

```
5'- aucccaccugggacaucuccuagucucccguugucacuggucuuaagagggcagaagaccagcuggacuggggacuaggagauaaccucccagaagugcuccuggacaacc   -3'   obs
    aucccaccugggacaucuccuagucucccguugucacuggucuuaagagggcagaagaccagcuggacuggggacuaggagauaaccucccagaagugcuccuggacaacc         exp
    .((((((((((((.((((((((((((((.(((....((((((((..(....)..))))))))...))).))))))))))))))....))))))..))).....))).....   reads   mm   sample
    ....................uagucucccguugucacuggu......................................................................   4       0    seq
    ....................uagucucccguugucacugguc.....................................................................   3       0    seq
    ....................uagucucccguugucacuggucu....................................................................   10      0    seq
    ....................uagucucccguugucacuggucuu...................................................................   2       0    seq
    .........................................................accagcuggacuggggacuag.................................   2       0    seq
```
